# Soil Water Status Monitoring System with Proximal Low-Cost Sensors and LoRa Technology for Smart Water Irrigation in Woody Crops

**DOI:** 10.3390/s24248104

**Published:** 2024-12-19

**Authors:** Jorge Dafonte, Miguel Ángel González, Enrique Comesaña, María Teresa Teijeiro, Javier J. Cancela

**Affiliations:** 1 PROEPLA, Higher Polytechnic School of Engineering, Campus Terra, Universidade de Santiago de Compostela, 27002 Lugo, Spain; mteresa.teijeiro@usc.es (M.T.T.) javierjose.cancela@usc.es (J.J.C.); 2 BioMODEM, Higher Polytechnic School of Engineering, Campus Terra, Universidade de Santiago de Compostela, 27002 Lugo, Spain; miguelangel.gonzalez@usc.es; 3 Department of Electronics and Computing, Higher Polytechnic School of Engineering, Campus Terra, Universidade de Santiago de Compostela, 27002 Lugo, Spain; e.comesana@usc.es

**Keywords:** agricultural water management, soil moisture, ESP32, LoRa, smart agriculture, Arduino IDE

## Abstract

Weather and soil water dictate farm operations such as irrigation scheduling. Low-cost and open-source agricultural monitoring stations are an emerging alternative to commercially available monitoring stations because they are often built from components using open-source, do-it-yourself (DIY) platforms and technologies. For irrigation management in an experimental vineyard located in Quiroga (Lugo, Spain), we faced the challenge of installing a low-cost environmental and soil parameter monitoring station composed of several nodes measuring air temperature and relative humidity, soil temperature, soil matric potential, and soil water content. Commercial solutions were either too expensive or did not meet our needs. This challenge led us to design the low-cost sensor system that fulfilled our requirements. This node is based on the ESP32 chip, and communication between the nodes and the gateway is carried out by LoRa technology. The gateway is also based on the ESP32 chip. The gateway uploads the data to an FTP server using a Wi-Fi connection with a 4G router while simultaneously storing the data on a memory card. The programming of the code for the nodes and the gateway is performed using the Arduino IDE. The equipment developed is proven to be effective and for managing vineyard irrigation based on the built-in sensors, with replicable results. It is, however, essential to calibrate the capacitive sensors for measuring soil water content in each soil type in order to enhance their ability to produce reliable results. In addition, the limits marking the beginning and end of irrigation tasks must be adjusted to local conditions and according to the producer’s specific vineyard objectives.

## 1. Introduction

In modern agriculture, the use of innovative technologies has become a necessity to improve efficiency, productivity, and sustainability. The use of sensors to increase efficiency is characteristic of Agriculture 4.0. In the context of climate change, managing water more efficiently has become essential. A key tool for improving water use in agriculture, particularly irrigation, is the Internet of Things (IoT) [1] and connected sensor networks (WSNs) [2]. These technologies can transform agriculture into a more efficient, sustainable, and productive activity. The combination of connected devices, sensors, and data analytics can revolutionize crop management, enabling precise monitoring and control of environmental conditions and crop yields.

The term IoT in agriculture refers to a network in which physical components (such as animals and plants), environmental elements, production tools, and various virtual “objects” in the agricultural system are connected to the Internet through the perception of agricultural information, with equipment under certain protocols to perform information exchange and communication [3]. IoT networks are generally divided into three layers: the perception layer, the transport layer, and the application layer.

The authors of [4] discuss the challenges facing agriculture and how the IoT can help producers optimize the resources used thanks to advances in small devices with low energy consumption that are able to collect data in the field. The IoT can then collect the information from these devices and apply resources (water, fertilizers, pesticides, etc.) only where necessary. This is closely linked to the concept of precision agriculture. The application of monitoring and automation can make agricultural production more sustainable and more efficient, as indicated by [5].

According to [4], the biggest challenges facing digitization in agriculture can be classified into communication problems, energy management, the heterogeneity of data and devices, data management, and generic platforms. In our opinion, this last challenge is one of the most complicated, since, in some cases, farmers’ lack of education and training can make it difficult to implement IoT networks in their farms. To address this issue, there are platforms such as mySense [6] that provide a set of free tools based on the do-it-yourself (DIY) concept and enable the use of low-cost platforms (Arduino, ESP32, Raspberry Pi, etc.) to quickly prototype and complete monitoring applications, enabling the rapid deployment of low-cost, integrated, and transparent technologies and thus increasing smart agriculture adoption.

A microcontroller unit (MCU) is an integrated electronic circuit (IC) that contains several processing units (CPUs) along with memory and programable input–output peripherals. MCUs are digital ICs designed for embedded applications, and they usually feature a limited set of instructions or operations that they can perform, making them suitable to create low-cost, low-power, and size-constrained control systems [7]. Popular examples of microcontrollers are the ATMega328 or the ATmega2560 from Microchip Technology Inc. (Chandler, AZ, USA) used in the Arduino development boards (Uno, Nano, and Mega). MCUs are the simplest form of a System-on-a-Chip (SoC) device. SoCs consist of the encapsulation of various computing units and other functionalities on the same IC, such as communication systems using radio frequency (RF) circuits or different kinds of sensors to measure physical or chemical magnitudes [5]. An example of an SoC is the ESP32 from Espressif Systems (Shanghai, China), which includes an Xtensa LX6 microprocessor, and Wi-Fi and Bluetooth connectivity, as well as different digital communication buses, data encryption functionalities, and circuitry for analog to digital signal conversion.

LoRa (Long Range) technology is a long-range (2–5 km in urban areas and 15 km in open areas), narrowband (up to 500 kHz) RF communication standard from the LoRa Alliance. Communication modules based on the LoRa standard, a type of signal modulation developed for IoT applications, enable the construction of low-cost and low-power-consumption systems, which are ideal for applications that require reliable, long-range data transmission, such as agricultural monitoring over large areas. Such IoT applications are known as low-power (LP) wide-area networks (LPWANs). The LoRa modulation technique is based on spread spectrum modulation derived from chirp technology [6]. Spread spectrum modulation involves generating signals with the widest possible spectrum over a fixed period of time, reducing the need for very high-quality factor (or low bandwidth) filtering, allowing for low selectivity and, therefore, low-cost communication systems. LoRa works in license-free sub-gigahertz radio frequency bands, such as EU868 (863–870/873 MHz) and EU433 (433.05–434.79 MHz) in Europe, and LP sensor networks can be designed and deployed as long as the devices emit signals below the maximum allowed power (25 mW or 14 dBm) and maintain a duty cycle below 10% (6 min of emission/reception per hour). In EU868/EU433, the transmission bitrates range from 290 to 5470 bits per second [8].

Previous research has explored the application of IoT devices employing LoRa data transmission in agriculture and irrigation scenarios in Spain. For example, ref. [9] developed an irrigation management system based on weather predictions using buried nodes, while [10] focused on designing an irrigation system with solenoids and flow meters in a pressurized irrigation setup with LoRa data transmission. From a network perspective, LoRa only provides a physical-layer method for wireless transport, such as via a transceiver chip, and lacks the necessary network protocols to manage data traffic and endpoint devices. There are several technologies available that can be connected to an LPWAN (low-power wide-area network) to accommodate for this, including Sigfox, LoRaWAN, and NB-IoT, as detailed in [11]. Looking ahead, the primary direction for agricultural sensor networks will be LPWAN (represented by the combination of LoRa and NB-IoT), which, when complemented by 4G and 5G technologies, will enable the transmission of large files.

For example, WSNs can collect real-time data on variables such as temperature, soil moisture, soil water status (matric potential and water content), and plant status [12]. These data are then transmitted via wireless networks to cloud analytics platforms, where they are processed and reports and recommendations are generated.

With these data, farmers can make decisions based on accurate and timely information. They can optimize irrigation [13], adjust the amount and timing of fertilizer application, prevent diseases and pests [14], and improve energy efficiency in agriculture [15]. The importance of knowing the total soil water available for vineyards, together with atmospheric demand (i.e., evapotranspiration rate), allows producers to determine the exact amount of water to be applied, as well as the most appropriate time for irrigation. Low-cost WSNs represent a competitive advantage for farmers by moving measurement points to the local area and by reducing costs and managing scarce water resources in a sustainable way. The high spatial variability that can occur in any plot can be captured by the placement of sensors at strategic points in the plot, although the inclusion of a high number of sensors does not guarantee an improved prediction of irrigation demand [16].

The integration of low-cost sensor data into decision-support systems, utilizing either simple algorithms or advanced AI (artificial intelligence), has driven significant advancements in the development of sensors and nodes for irrigation management in crops like grape vines [17]. The goal is to be able to use these sensor networks to control the opening and closing of irrigation valves [18], making it easier for farmers to manage irrigation.

The cost of a commercial environmental data-acquisition system able to detect the temperature and relative humidity of the air, precipitation, shortwave incident solar radiation, and wind speed and with the capacity to send data remotely is in the order of EUR 2000–3000 (excluding taxes).

The objective of this work is the design and programming of a node with a data-acquisition system and with low-cost sensors for measuring the air temperature and relative humidity, soil matric potential, soil water content, and soil temperature and which has the capacity to transmit data between the nodes and the concentrator using LoRa technology. The concentrator will be equipped with data storage capacity and the ability to upload data to the cloud to monitor the effects of different treatments on a vineyard. The system will be used to manage irrigation efficiently, based on the readings of various soil water sensors and the study of variations in environmental conditions below and above the plant canopy. In addition, the efficiency of applying the same dose of irrigation at different frequencies will be evaluated. To this end, the aim is to measure temperature and relative humidity of the air under and above the plant canopy, soil temperature, and soil water status.

## 2. Location and Experiments

The equipment was designed to monitor a 1.3 ha commercial vineyard of the Mencía variety trained on trellises, located in Quiroga (Lugo) (42°28′16.38″ N, 7°15′1.70″ W). From 2021 to 2023, several drip irrigation treatments were implemented: a daily irrigation treatment (T1) starting from the pea-sized grain phenological stage and covering 30% of the reference evapotranspiration (ET_o_). In addition, a non-irrigated treatment (T0) was incorporated as a control. In treatments T0 and T1, a second study variable was implemented, incorporating slate remains into the vineyard line, to evaluate its effect on the control of vegetation in the line and the microclimate in the cluster area [19]. Each treatment consists of four randomized repetitions with seven control plants.

**Figure 1:**
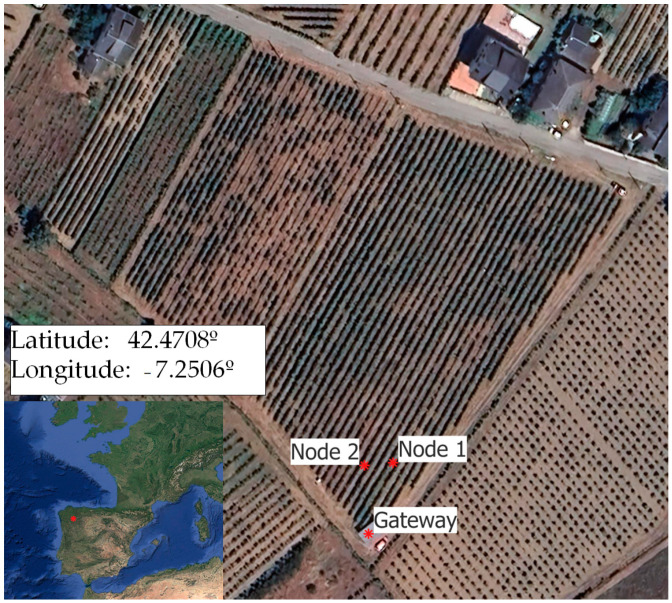
Experimental site location.

In this initial phase, two nodes were installed: node 1 (T1, daily irrigation without slate) and node 2 (T0, rainfed without slate) (Figure 1).

In the commercial vineyard, the parameters measured were air temperature and relative humidity, soil temperature, soil matric potential, and soil water content (sensor description in Section 3). Moreover, stainless steel rods were installed at a 15 cm depth (4 points) to obtain discontinuous measures of soil water content (TDR) and calibrate capacitive soil water content sensors. Soil water content was measured twice per week using a TDR-100 (Campbell scientific, Inc., Logan, UT, USA) from May to October 2023 (28 days of measurement). For this experiment, the low-cost capacitive sensors provide readings from a depth range of 0 to 10 cm. The soil temperature sensor, which has a length of 3 cm, was installed at a depth of 5 cm.

In a parallel laboratory study conducted using sand-filled pots, commercial sensors (Teros 12, Meter Group, Inc., Pullman, WA, USA) were used to evaluate the performance of low-cost capacitive soil water content sensors (SEN 0308, DFRobot, Shanghai, China) and to examine their response to variations in soil temperature. For this experiment, the sand was completely saturated, and the sensors’ response was measured as the sample dried. For this specific test, the data obtained from the Teros 12 sensors relate to measurements taken at a depth of 5.5 cm. Conversely, the low-cost capacitive sensor provides readings from a depth range of 0 to 10 cm. The soil temperature sensor, which has a length of 3 cm, was installed at a depth of 5 cm.

## 3. Design of System for Measuring, Recording, and Transmitting Data

The measurement system consists of nodes that communicate with a hub using LoRa technology. The hub stores the data on a memory card and sends the data through a connection to a Wi-Fi network generated by a router.

**Figure 2:**
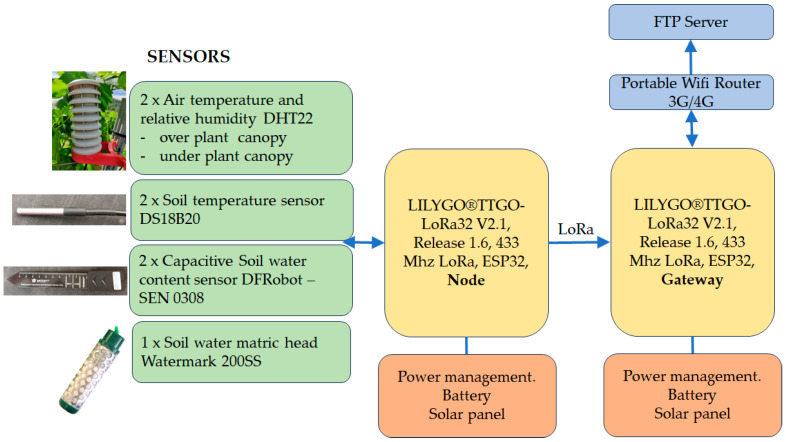
Field node architecture of wireless sensor network.

The sensors used in each node are two air temperature and relative humidity measurement sensors (DHT22, DFRobot, Shanghai, China), two soil temperature measurement sensors (DS18B20, DFRobot, China), a Model 200SS watermark sensor (Irrometer Company, Inc., Riverside, CA, USA) for measuring the matric potential of water in the soil, and two capacitive sensors for measuring soil water content (SEN 0308, DFRobot, Shanghai, China) (Figure 2). All the sensors used, except for the soil water potential measurement sensor, are modular devices that, in addition to the transducer element itself, incorporate complex signal conditioning, linearization, and communication with the microcontroller through data transmission protocols.

### 3.1. Sensors

The DHT22 air temperature and humidity sensor is a low-cost digital sensor that includes a capacitive humidity sensor and an NTC thermistor to measure air temperature and relative humidity. It outputs a digital signal to the digital data pin using a single-wire transmission bus. The sensor operates in the voltage range of 3–5 V. The characteristics are as follows: a temperature range of −40 °C to 80 °C; an accuracy within ±0.5 °C and ±1 °C (maximum) in adverse conditions; a relative humidity range of 0% to 99.9%; and an accuracy within ±2%RH at a temperature of 25 °C. It is mounted on a board with a pull-up resistor and filtering capacitor. Each of the sensors is covered with a radiation shield (Figure 2).

The DS18B20 soil temperature sensor (DFRobot, Shanghai, China) is an encapsulated and programmable sensor with a single cable; its connection to the board requires a single data pin, along with a 4.7 k Ω pull-up resistor.

The DS18B20 communicates over a one-wire bus that allows a single digital port to be used to measure multiple sensors. Each of the sensors has a unique 64-bit hex address from the factory that must be entered into the program code. This sensor can measure temperatures between −55 °C and 125 °C, with a programmable resolution between 9 and 12 bits.

**Figure 3:**
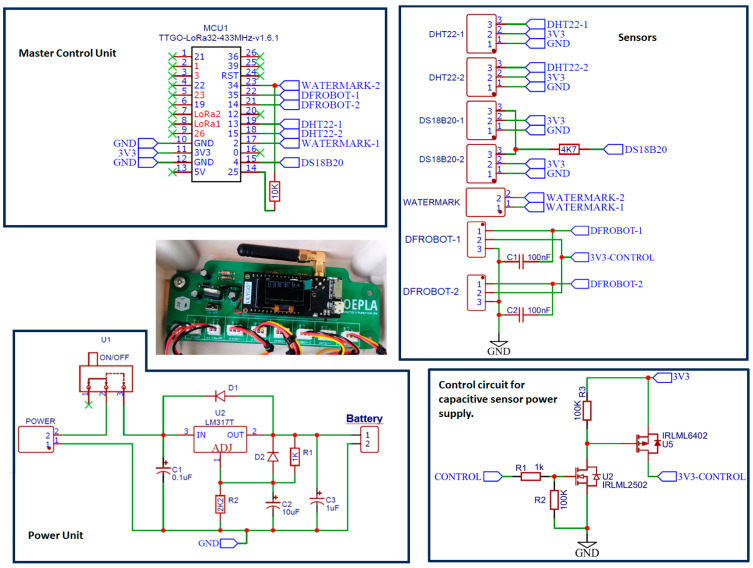
Schematic of circuit node.

The Model 200SS (Irrometer Company, Inc., Riverside, CA, USA) sensor was used to measure the matric potential of soil water. This sensor can measure soil water matrix potentials down to −200 KPa. The transducer element consists of a pair of highly corrosion-resistant electrodes embedded in a granular matrix. A current is applied to the sensor to obtain a resistance value, and by means of regression equations with the resistance and temperature data of the soil, the value of the matric potential in kPa is obtained. Another important feature for the reliability of the reading is the presence of internally installed gypsum, which provides a buffering effect against the potential effect of salinity levels that may exist in the soil. These types of sensors must be used with alternating polarity to avoid the build-up of a charge that compensates for the reading and degrades the electrodes over time. The chosen option was a short pseudo-AC pulse. Alternation can be achieved by using two digital pins in which one is grounded (low state) and the other provides power (high state). After a programmed time, the assignment of states on the two pins is reversed, thus achieving a rectangular wave that, for practical purposes, behaves as an alternating current wave in the sensor. This leaves the sensor with no built-up potential, immediately ready for further readings. Signal conditioning is carried out by means of a voltage divider. Excitation should not exceed 50 ms in total, and measurements should be made within 100 μs. This sensor requires two digital outputs to create the pseudo-alternating current and an analog input to measure the voltage and transform it into resistance (Figure 3).

The capacitive soil water content sensor, a Model DFRobot SEN0308 (DFRobot, Shanghai, China), is a sensor with an analog output. The module uses a TL555I CMOS timer to create a clock of 1.5 MHz. A peak voltage detector converts the TL555I’s waveform into a DC voltage that can read the ADC input from a microcontroller. When the probe is exposed to moisture, it affects the capacitance of the circuit, which, in turn, affects the maximum amplitude of the signal and therefore the DC voltage output that is being monitored by the microcontroller. Higher humidity implies lower voltage output [20].

The sensor can operate in a power range of 3.3–5.5 V, and like all capacitive sensors, it requires a specific calibration for each type of soil.

This sensor has an approximate consumption of 6.6 mA at 3.3 V; this consumption is continuous, so it causes a decrease in battery life. To solve this problem, a circuit was implemented with two MOSFET transistors activated by a control pin; the schematic of the power supply to the two capacitive sensors can be seen in Figure 3.

In addition, ESP32 ADC can be sensitive to noise, leading to large discrepancies in ADC readings (Figure 3). To minimize noise, users may connect a 0.1 μ capacitor to the ADC input pad in use. Multisampling may also be used to further mitigate the effects of noise; in this case, the average of 10 measurements is used.

### 3.2. Microcontroller

The ESP32 is a low-power, high-throughput SoC, designed especially for IoT applications. It has a wide range of features, including Wi-Fi and Bluetooth connectivity, the ability to run programs autonomously, and many input and output pins for connecting sensors and actuators. In this project, the TTGO LoRa32 T3_V1.6.1 development board (LILYGO Shenzhen Xinyuan Electronic Technology Co., Ltd., Shenzhen, China) was used. This board includes the ESP32 PICO-D4, a flash memory of 4 MB, a 0.96-inch OLED display, TF/microSD card circuitry and card slot, a power input/lithium battery management interface, a Semtech SX1278 (Semtech Corp., Camarillo, CA, USA) LoRa transceiver, and an SMA antenna connector. It also includes a USB to UART interface that allows the use of programming environments such as Arduino, micropython, PlatformIO, or other standard Integrated Development Environments (IDEs). As for the ESP32, it provides a 12-bit digital analog converter, 10 analog inputs, and 17 digital inputs/outputs accessible in the TTGO Lora32 development board.

One reason for choosing this board is that it met the requirements for the design, meaning additional peripherals are not needed. Its low power consumption compared to other boards such as Arduino Uno or Arduino Mega (only 0.2 μA in deep sleep mode) is another reason for its selection.

The programming language used was C++, using the Arduino IDE.

### 3.3. Data Transmission

For data transmission between the node and the concentrator, LoRa is used, using the frequency band EU433. Although it has a lower data transmission capacity compared to the next available range of EU868, it was decided to use this band because it has a lower signal attenuation. In the case of buried nodes, as described by [9], propagation is reportedly better at a frequency of 433 MHz due to the higher transmitted power and lower propagation losses. It was decided not to use LoRaWAN due to its greater complexity, and for the number of nodes that will be managed, this protocol is not necessary. Another added difficulty is that the LoRaWAN library, available in the Arduino ecosystem, would have to be modified, as it currently does not support 433 MHz communications. The device network connection scheme is star-shaped.

One drawback of not using the LoRaWAN protocol is that the transmitted data are unencrypted, i.e., open. In addition, to solve the possible problem of interference or corrupted data transmission, the enable Crc function of the Sandeepmistry LoRa library (https://github.com/sandeepmistry/arduino-LoRa, accessed on 6 November 2024) was introduced, and a control tag with the name of the node was also added.

The data file is sent from the hub to a 4G wireless router connected to the Internet through a Wi-Fi network.

### 3.4. Node

As indicated above, each node consists of a TTGO LoRa32 T3_V1.6.1 board, with an ESP32 chip, and records measurements from two DHT22 air temperature and humidity sensors, two DS18B20 soil temperature sensors, a Watermark sensor, and two capacitive sensors for measuring soil water content data. Data are recorded every five minutes.

**Figure 4:**
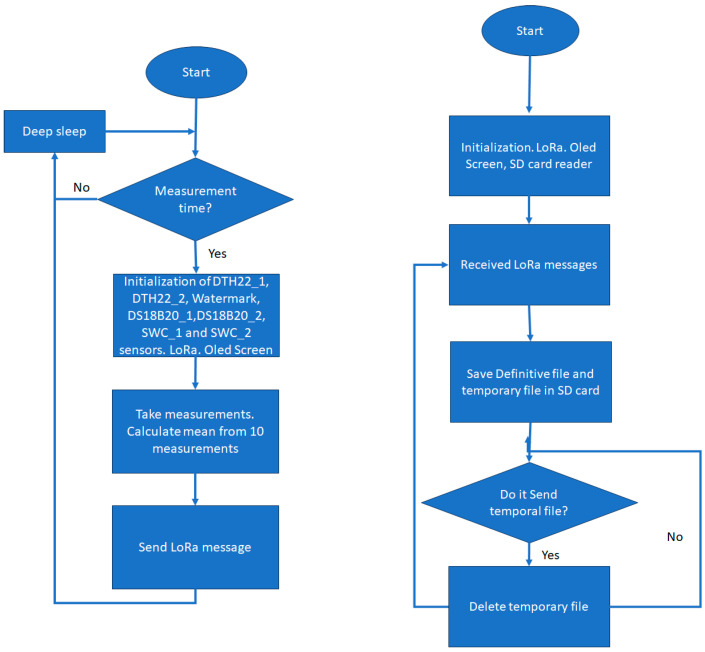
Flowchart of node programming (**left**) and flowchart of gateway programming (**right**).

The power module consists of a 12 V battery, a charge regulator, and a solar panel. The sensors are powered by a 5 V output from the charge regulator, then pass through a voltage regulator to go from 5 V to 3.3 V. A MOSFET transistor is also installed that allows the power supply of the sensors to be controlled with a logic output from the microcontroller, avoiding energy consumption during the time between measurements. To reduce power consumption at times of non-use, the microprocessor can enter deep sleep, for which all the measurement instructions in the Arduino IDE are in the setup mode and not in the loop mode (Figure 4).

After the microprocessor emerges from deep sleep, a buffer time is established prior to the measurements to allow the stabilization of the response of the sensors. In this case, a warm-up time of 30 s is used.

Date and time information is not stored on the node, nor are the data stored.

**Figure 5:**
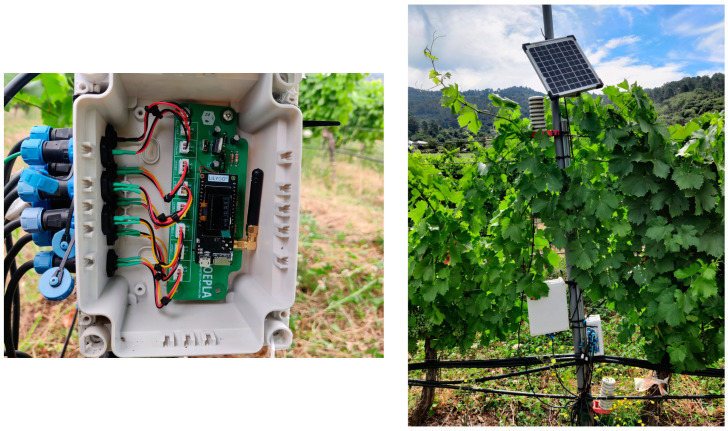
Node with board, connections, and microcontroller (**left**) and node installed in field (**right**).

The sensors are connected to the box where the electronics are located by means of IP68 waterproof SP13-type aviation connectors. Figure 5 shows photographs of the nodes installed in the plot.

### 3.5. Gateway

The hub or gateway is based on the same board as the node equipment, but without any sensor and with data recording on a microSD card in two files, a permanent file and a temporary file that is deleted once the data have been sent to the network. When the gateway receives a LoRa message from the nodes, it adds a date and time tag, which is fetched from the hora.roa.es NTP server. At a frequency set in the code, the temporary data file is uploaded to an ftp server with the data from the temporary data file, and is then locally deleted once sent (Figure 4).

The hub connects to the network created by a 4G wireless router to receive the sensor data and send the files of the recorded data.

The power supply to the hub is provided by a 230 V AC to 12 V DC transformer, which feeds a charge regulator that gives a 5 V output and a battery to ensure power supply in the event of a power outage. The option of connecting it to the electricity grid was chosen because this equipment is inside the irrigation pumping station building that has an electricity supply, and the concentrator has a higher electricity consumption than the nodes due to the Wi-Fi network and 4G modem.

## 4. Results and Discussion

**Figure 6:**
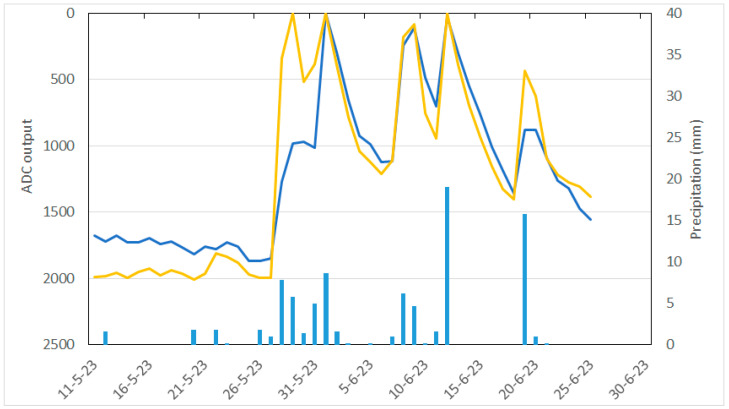
Daily average data of the output of the analog digital converter of the two capacitive sensors of soil water content (blue line capacitive sensor 1 and yellow line capacitive sensor 2) of node 1 and daily precipitation (blue bars).

As an example of the final results, Figure 6 shows the daily average soil water content in node 1 (rainfed treatment). It should be taken into account that the number that appears on the abscissa axis represents the output of the digital analog converter, which is the ESP32, whose converter has a resolution of 12 bits, and 0 is 0 V and 4095 is 3.3 V.

Regarding the transmission of data by LoRa, the two nodes in the measurement period from 10 May 2023 to 13 December 2023 theoretically received 62,496 messages, and as nine parameters were measured (temperature and relative humidity of the air below and above the vegetation cover, soil temperature at two points, soil water potential and water content in the soil at two points), this corresponds to 562,464 data points. After analyzing the messages received, the percentage of lost messages was 0.02%, while the percentage of messages with errors was 0.63%. This indicates the high reliability of receiving messages at the gateway with LoRa technology.

**Figure 7:**
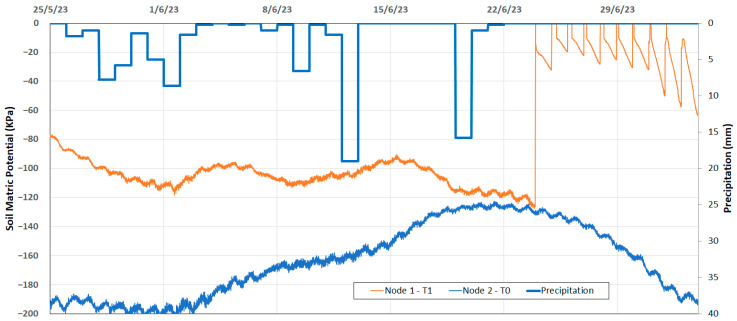
The 10 min data of matric potential in kPa at node 1 and 2 and daily precipitation (blue bars).

Finally, Figure 7 shows the soil matric potential data measured with the watermark sensor; the average measurements were stable. As shown in [16], increasing the number of granular matrix sensors in the sensor set does not increase the potential of reduced within-field variability. Each node includes one watermark sensor, which is enough for proper irrigation management. As drip irrigation was initiated on 24 June 2023, as shown in Figure 7, the soil matric potential in the T1 treatment exhibited a relatively stable trend, contrasting with the T0 treatment where the matric potential showed a strong correlation with precipitation. However, Watermark sensor data from the T0 treatment became unreliable after 4 July 2023 due to the sensor’s measurement limit of −200 kPa [21].

We want to highlight the low cost of a node with two data sensors for measuring the temperature and relative humidity of the air, two soil temperature sensors, two capacitive sensors for measuring the soil water content, and a watermark sensor for measuring the soil matric potential. The cost of all these sensors considering wiring, connectors, and installation, in addition to the development board and with power supply through a solar panel, is approximately EUR 300 (excluding taxes), which is much cheaper than a commercial solution, which has a cost of around EUR 1500–2500. Several studies have used these types of matric potential sensors [22] and capacitive sensors to measure water content in the soil [23]. One issue to keep in mind is the importance of obtaining specific calibration curves for each type of soil.

The calibration of the capacitive sensors was performed with respect to the measurement of soil water content (θ) measured with the time-domain reflectometer (TDR) model TDR100 (Campbell Scientific, Logan, UT, USA), which operates in the field using PCTDR software (V3.0, Campbell Scientific, Logan, UT, USA). Measurements were performed using a flexible reading head designed by [24], with θ calculated using the equation by [25]. For this purpose, stainless steel rods were buried at a 15 cm depth.

**Figure 8:**
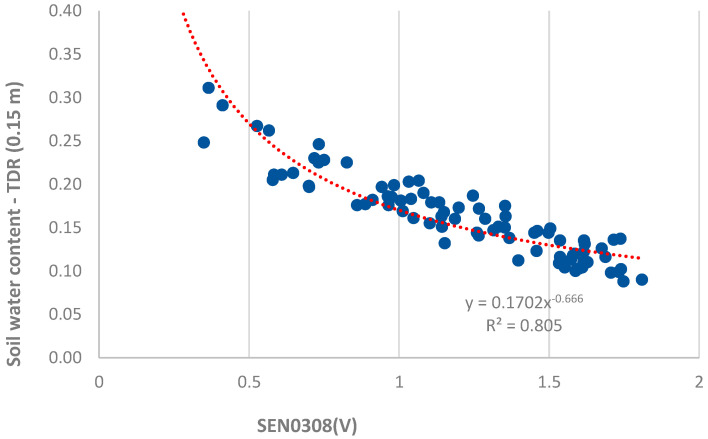
A calibration curve of the voltage output of the capacitive sensor against the soil water content estimated with TDR for vineyard soil, raw data points (dots) and fitted model (dashed line).

**Figure 9:**
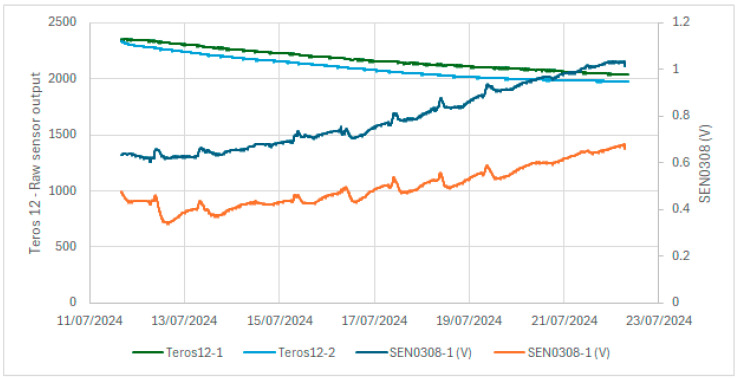
Measurements of Teros 12 sensors and DFRobot SEN0308 sensors in sand after saturation.

It can be seen in Figure 8 that the location of the four installed sensors (two sensors in each node) leads to significant variability in the data collected, obtaining an R² of 0.805 with a potential type of equation. In another work using a similar sensor (SEN0193 DFRobot, Shanghai, China) [26], it is pointed out that this type of sensor gives better results when calibration is performed for each individual sensor (all sensors had an R^2^ above 0.94). A dependence of the sensor outputs on the soil temperature was also detected, as also pointed out in [26], but its effects are small, which also highlights that this type of sensor does not have a comparable accuracy to commercial sensors. Figure 6 and Figure 9 show a differing response among the low-cost SEN0308 sensors, a discrepancy also observed in [26]. In contrast, the difference between the two analyzed Teros 12 sensors was much smaller, as shown in Figure 9. In our opinion, it is challenging to significantly improve the R² obtained due to the variability observed between the measurements of individual sensors for the same soil water content.

**Figure 10:**
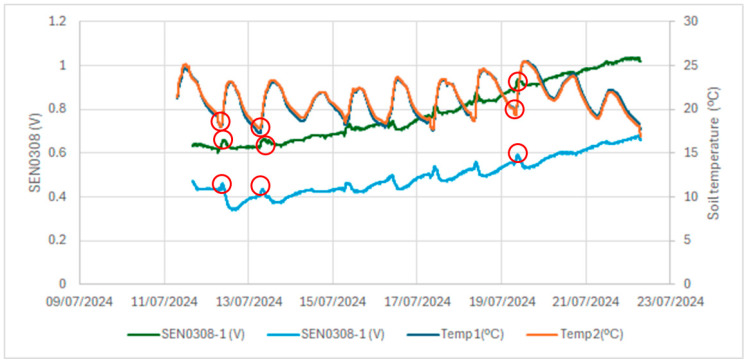
DFRobot SEN0308 sensor measurements in sand after saturation and soil temperature. Red circles show the influence of soil temperature on the output of SEN0308.

In a laboratory investigation, a pot of sand was used. The responses of two low-cost capacitive sensors were compared to those of two commercial Teros 12 sensors. Figure 9 shows the results of the raw output of the Teros 12; the Teros 12 microprocessor measures the charge time and outputs a raw value based on the substrate dielectric permittivity. There was less difference between sensors and less noise in the case of the Teros 12 sensors. In the case of the low-cost capacitive sensors, there is a greater difference between the sensors and a greater noise level. In our opinion, one of the main drawbacks of the low-cost sensors is the existing difference in the output values of the two tested sensors under the same conditions. This difference is also observed in the field (Figure 6), where the dispersion of the measurements increased, as reflected in a not-so-high R² value (0.805) in the calibration curve of the low-cost capacitive sensors when using all low-cost sensors in the field (Figure 8). Figure 10 compares the voltage output of the DFRobot SEN0308 capacitive sensors with the soil temperature measurements and shows some dependency of the values on the soil temperature. The red circles in Figure 10 mark points where the soil temperature exerts an inverse influence on the sensor output, with a discernible time lag. This temperature effect is more pronounced at lower outputs of the low-cost capacitive sensor, corresponding to higher soil moisture content, as illustrated in the calibration curve from the field experiment (Figure 8). This temperature dependence is not shown in the Teros 12 sensors, as can be seen in Figure 9.

Despite the existing differences in the outputs of the different low-cost capacitive sensors for the same conditions, in our opinion, it is a very interesting alternative, as the price of low-cost sensors is around EUR 15, and the cost of a Teros 12 sensor is around EUR 300. Therefore, with the cost of a single Teros 12 sensor, we can install 20 low-cost capacitive sensors, which would give us more information about the spatial variability in soil water status, although not with the precision that the Teros 12 provides.

As [27] highlight in their research on smart agriculture adoption in Brazil, farmers’ educational background and technical proficiency to effectively utilize these advanced agricultural practices are a significant barrier. We contend that the complexity involved in programming and calibrating sensors presents an additional hurdle. However, this technological gap offers a lucrative opportunity for domestic tech firms to provide tailored solutions and support services, empowering farmers to embrace smart agriculture.

The implementation of this crop water status monitoring technology has the potential to optimize water management, especially in developing countries. The cost-effectiveness of these devices, relative to conventional commercial options, is a compelling factor. Moreover, LoRa technology offers a promising solution for data acquisition from remote field installations. Nevertheless, the technological proficiency required for system operation remains a limiting factor, as previously discussed.

## 5. Conclusions and Future Developments

The use of low-cost microcontrollers and sensors can greatly reduce the cost of investing in smart agriculture networks, facilitating the incorporation of technology by farmers and therefore the adoption of the Agriculture 4.0 model. Thus, the main advantages of our proposed node are the cost and the flexibility of the system, since the hardware system and code can be modified to meet the needs of each scenario. The existence of a vast amount of information freely available on the Internet concerning low-cost sensors and this type of MCU can also be highlighted as an advantage, which can facilitate the learning and programming of the code.

Regarding drawbacks, this type of MCU does not have a user-friendly programming environment, which is an issue for operators with minimal knowledge of programming and electronics. In addition, the accuracy of the capacitive sensors of soil water content is lower compared to commercial alternatives (i.e., the TEROS 12), and the same applies to the variability between sensors.

In this section, a series of future improvements are proposed. The first would be to make the communication between the node and the gateway bidirectional, rather than unidirectional, allowing the measurement order to come from the gateway. This is because the ESP32′s internal clock is not precise over the long term, so the period between measurements may deviate over time. Beyond enabling the synchronization of data-acquisition times between nodes and gateways, bidirectional communication offers the potential for future applications such as the creation of control nodes to manage irrigation systems, including pump operation and solenoid valve control for precise irrigation sector management.

A second future perspective would be to integrate these nodes with a platform such as MySense [6] for cloud storage, management, and processing of the received data. In the future, there could be a level of integration with a smart gateway capable of managing the irrigation system based on the information received by a decision-support system (DSS).

A third perspective would be to integrate a connection between the data management platform and a DSS for the automatic programming of the irrigation system based on the data obtained by the sensors. An example of such a DSS is IrriDesk [28], which is oriented towards the management of irrigation in commercial vineyards.

## Data Availability

Sketches of the Arduino IDE for the sender device are available at https://doi.org/10.5281/zenodo.13983058, and the dataset used in this research is available upon reasonable request to any of the authors of this research article.
